# Thermodynamic analysis of DNA binding by a *Bacillus* single stranded DNA binding protein

**DOI:** 10.1186/1471-2091-13-10

**Published:** 2012-06-14

**Authors:** Esther E Biswas-Fiss, Jirayu Kukiratirat, Subhasis B Biswas

**Affiliations:** 1 Department of Molecular Biology, School of Osteopathic Medicine & Graduate School of Biomedical Sciences, University of Medicine & Dentistry of New Jersey, Stratford, NJ 08084, USA; 2 Department of Bioscience Technologies, Jefferson School of Health Professions, Thomas Jefferson University, Philadelphia, PA 19107, USA

**Keywords:** Single-stranded DNA binding protein (SSB), DNA replication, Fluorescence anisotropy, ssDNA binding, Protein-DNA complex

## Abstract

**Background:**

Single-stranded DNA binding proteins (SSB) are essential for DNA replication, repair, and recombination in all organisms. SSB works in concert with a variety of DNA metabolizing enzymes such as DNA polymerase.

**Results:**

We have cloned and purified SSB from *Bacillus anthracis* (SSB_BA_). In the absence of DNA, at concentrations ≤100 μg/ml, SSB_BA_ did not form a stable tetramer and appeared to resemble bacteriophage T4 gene 32 protein. Fluorescence anisotropy studies demonstrated that SSB_BA_ bound ssDNA with high affinity comparable to other prokaryotic SSBs. Thermodynamic analysis indicated both hydrophobic and ionic contributions to ssDNA binding. FRET analysis of oligo(dT)_70_ binding suggested that SSB_BA_ forms a tetrameric assembly upon ssDNA binding. This report provides evidence of a bacterial SSB that utilizes a novel mechanism for DNA binding through the formation of a transient tetrameric structure.

**Conclusions:**

Unlike other prokaryotic SSB proteins, SSB_BA_ from *Bacillus anthracis* appeared to be monomeric at concentrations ≤100 μg/ml as determined by SE-HPLC. SSB_BA_ retained its ability to bind ssDNA with very high affinity, comparable to SSB proteins which are tetrameric. In the presence of a long ssDNA template, SSB_BA_ appears to form a transient tetrameric structure. Its unique structure appears to be due to the cumulative effect of multiple key amino acid changes in its sequence during evolution, leading to perturbation of stable dimer and tetramer formation. The structural features of SSB_BA_ could promote facile assembly and disassembly of the protein-DNA complex required in processes such as DNA replication.

## Background

Nearly all cellular nucleic acid transactions, including DNA replication, repair and recombination require the activity of a single stranded DNA binding protein (SSB) [1-7]. SSB proteins and are found throughout nature and their functional importance is underscored by their presence in prokaryotes, archaea, and eukaryotes including mammals [1]. Among its multifaceted roles, upon binding to ssDNA, SSB prevents the reformation of duplex DNA making it possible for other enzymes such as DNA polymerase to use ssDNA as substrate. In addition, the binding of SSB-type proteins protects the ssDNA molecules from attack by intracellular nucleases. Although not possessing intrinsic enzymatic activity in and of themselves, SSB proteins are known to influence the activities of many enzymes as well as to organize the multi-protein complexes required for processes such as DNA replication, recombination and DNA repair [8-11].

The function of SSB during DNA replication has been extensively studied in *E. coli*, which serves as the prototypical model system for prokaryotes and eukaryotes alike. In *E.* coli, the large nucleoprotein replication initiation complex is stabilized by single stranded DNA binding protein, following which DNA is unwound by the DnaB helicase protein. Efficient DNA unwinding activity of DnaB protein in progression of the replication fork in *E. coli* is strongly dependent on the continued action of a cognate SSB [12,13]. SSB works in concert with DnaB helicase, DNA primase, and DNA polymerase III holoenzyme during *E. coli* DNA replication [5,9,12,14,15]. Phage λ DNA replication requires the participation of host *E. coli* SSB as well [16,17]. In archaea and eukaryotes, its functional homolog, Replication Protein A (RPA), carries out the role of organizing and stabilizing the replisome during DNA replication [1,3,10,18-21].

Vital to its function in DNA metabolism is the structure of SSB. In the Gram-negative bacteria, SSB is homotetrameric, with each monomer contributing a single ssDNA-binding domain to the functional form. The eukaryotic RPA is composed of three subunits (RPA70, RPA32, and RPA14) and functions as a heterotrimer through the use of four ssDNA-binding domains [2,3,18].

Unlike *E. coli* SSB, single stranded DNA binding protein from bacteriophage T4, the gene 32 protein, is a monomer. T4 gene 32 protein can form multimers at high concentration induced by high salt and high pH [22]. Kim and Richardson demonstrated that the bacteriophage T7 SSB, the gene 2.5 protein, is a dimer [23]. The T7 gene 2.5 SSB appears to bind DNA as a dimer. The ssDNA binding affinities of both T4 and T7 SSBs are lower than that observed with *E. coli* SSB. Despite these differences, ssDNA binding of SSB proteins using OB fold-domains (oligosaccharide/oligonucleotide binding domains) appears to be universal throughout all systems described to date [1].

The *E. coli* SSB is highly cooperative in ssDNA binding that is influenced by salt concentration [24,25]. Recent studies indicate that SSB has at least two distinct modes of ssDNA binding [26]. The binding is modulated by monovalent salts. At very low salt concentration (<10 mM NaCl), SSB binds ssDNA using two of its four subunits in a highly cooperative manner and occludes only 35 nucleotides [(SSB)_35_ mode]. On the other hand, at higher salt concentrations (>200 mM NaCl), it binds to ssDNA using all four subunits and protects ~65 nucleotides [(SSB)_65_ mode]. It is not clear how the ssDNA binding is altered between 10 and 200 mM NaCl. Higher-order forms of SSB in ssDNA bound states, based on high resolution electron microscopic studies of SSB-ssDNA complex, have also been reported [27]. Chrysogelos and Griffith discovered that repeated freezing-thawing of *E. coli* SSB leads to the formation of unique strings of tetramers [28].

Gram-positive bacterial protein sequences do not form a monophyletic group, but are intermixed with plasmid and phage sequences [29,30]. Gene organization in these organisms can differ from that observed in Gram-negative *E. coli* and these organisms may contain multiple paralogues [31,32]. Sequence analysis indicated that Gram-positive SSBs have a highly conserved nearly-identical (>90% identity) N-terminal ssDNA binding as well as monomer-monomer interaction domains but they differ to some extent from the Gram-negative SSBs. We have investigated the structure and ssDNA binding of a Gram-positive bacillus SSB (SSB_BA_) in order to understand its mechanism of action of SSBs in these organisms. We present here a report of a Gram-positive SSB that utilizes a novel structural mechanism for protein-DNA interaction using a transient tetramer formation.

## Results

The single-stranded DNA binding protein ORF of *B. anthracis* (*BAS5326)* was identified by BLAST search of the annotated sequenced genome of *B. anthracis* Stern strain [33,34]. The ORF encodes a polypeptide of 172 amino acid residues with a predicted molecular weight of 19.2 kDa.

### Sequence analysis of SSB_BA_

**Figure 1:**
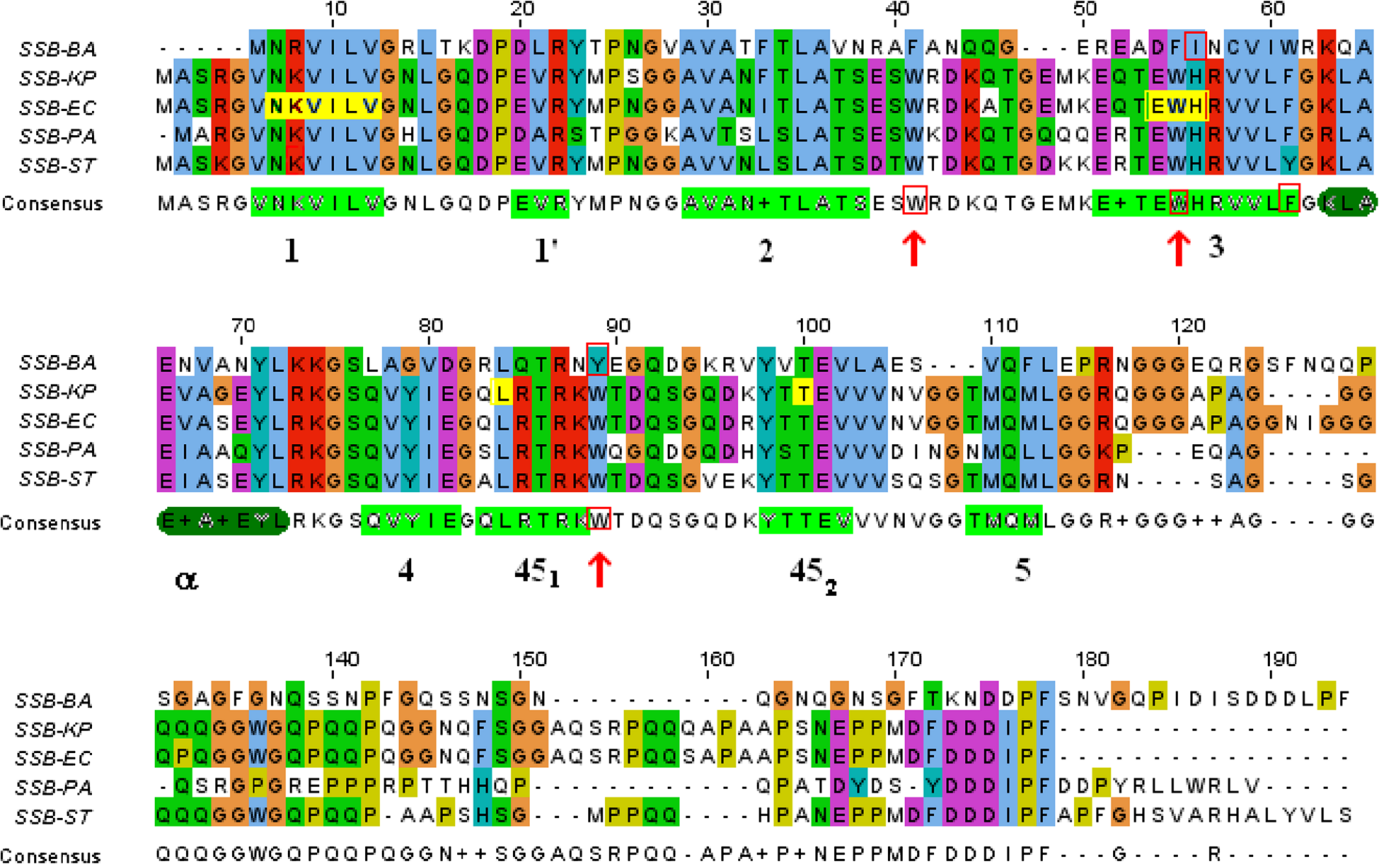
**Sequence alignment of SSB proteins.** Gram-positive and Gram-negative bacteria: ssDNA binding and protein-protein interactions domains of SSB proteins from Gram-positive *B. anthracis* (SSB-BA), and Gram-negative *E. coli* (SSB-EC), *Salmonella typhimurium* (SSB-ST), *Klebsiella pneumoniae* (SSB-KP), *Pseudomonas aeruginosa* (SSB-PA) were aligned using ClustalW2 program. The color coding is as follows: red, basic; blue hydrophobic; green, hydrophilic; orange, neutral; pink, acidic; and light green, proline. Notations of the secondary structures in SSB_BA_ are as defined by Murzin [38] with the β strands of the OB-fold labeled 1–5 (Light green box) and the α-helix (dark green box). Locations of the important hydrophobic tryptophans (in *E. coli*) are indicated by red up arrows. The residues in the monomer-monomer interface of the dimer in *E. coli* SSB sequence are indicated in yellow boxes. Changes of Gln77 →Leu and Gln111→Phe should destabilize dimer-dimer interaction important for the tetramer formation [39].

The amino acid sequence of the N-terminal ssDNA binding and protein-protein interaction domains responsible for dimer and tetramer formation of SSB_BA_ were compared with the sequences of a Gram-positive (*Bacillus anthracis*) and Gram-negative (*E. coli* and *Salmonella typhimurium*) SSB proteins. Multiple-alignment of these sequences, using ClustalW2, is shown in Figure 1. In general, a high degree of sequence homology among SSBs was observed only in the N-terminal two-thirds of the proteins. In the N-terminus two-third, sequence homology (identity + similarity) of SSB_BA_ was observed with Gram-positive SSBs and was estimated to be ~54%, whereas, it was ≥90% among Gram-negative SSBs. Amino acid residues 1–46 constitute the major portion of the SSB core domain and contain the OB DNA binding fold. The second region of homology observed was between residues 51–104 (Figure 1). A third area of homology was also observed in the last six residues with the sequence, DDDLPF, which corresponds to the acidic carboxy-terminal domain characteristic of eubacteria; this sequence is required for interaction with recombination, replication and repair machineries [35,36]. The C-terminal region lacked significant homology even among Gram-negative SSBs in the region between the residues 100–166 among these SSBs [37].

### Purification of SSB_BA_

**Figure 2:**
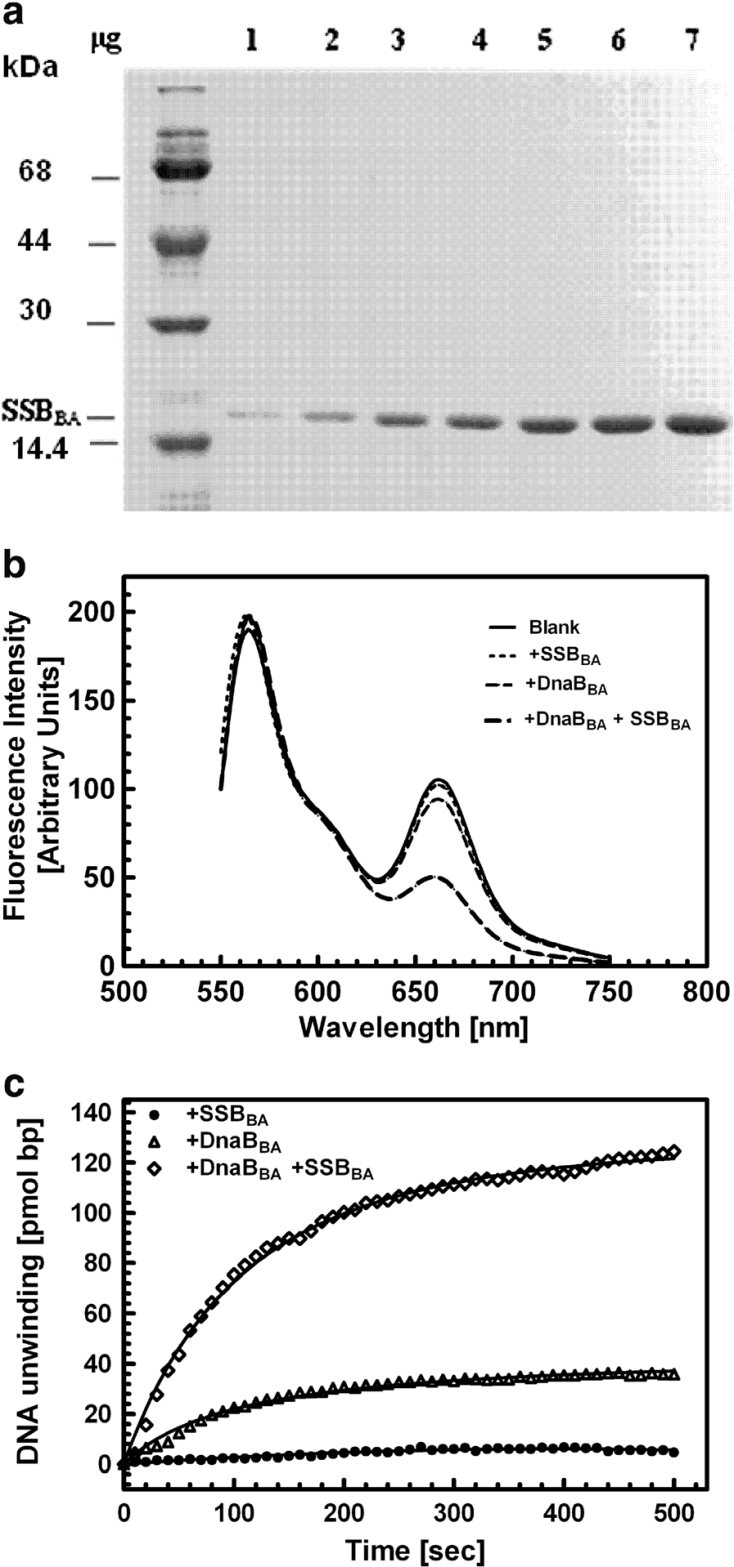
**SDS-PAGE and biological activity of purified SSB**_**BA**_**. (a)** SDS-PAGE analysis of SSB_BA_ (fraction V) used in this study. **(b)** FRET helicase analysis of DnaB_BA_ in the presence and absence of SSB_BA_: Emission spectra of the substrate (4.2 nM), after 15 min incubation with SSB_BA_ (3 μg/ml) DnaB_BA_ (0.5 μg/ml), and both SSB_BA_ and DnaB_BA_ at 37°C; **(c)** Kinetic analysis of helicase activity: The helicase substrate was rapidly mixed with indicated protein(s) and fluorescence emission at 662 ± 8 nm was recorded as a function of time for 500 s using a Slow Kinetic mode in PC1 spectrofluorometer using Vinci software (ISS Inc. Champaign, IL). Using FRET spectra of native and heat denatured substrate, we predetermined that 1% decrease in FRET is equivalent to ~3.1 pmol of nucleotide (or bp) unwinding of duplex DNA.

**Figure 3:**
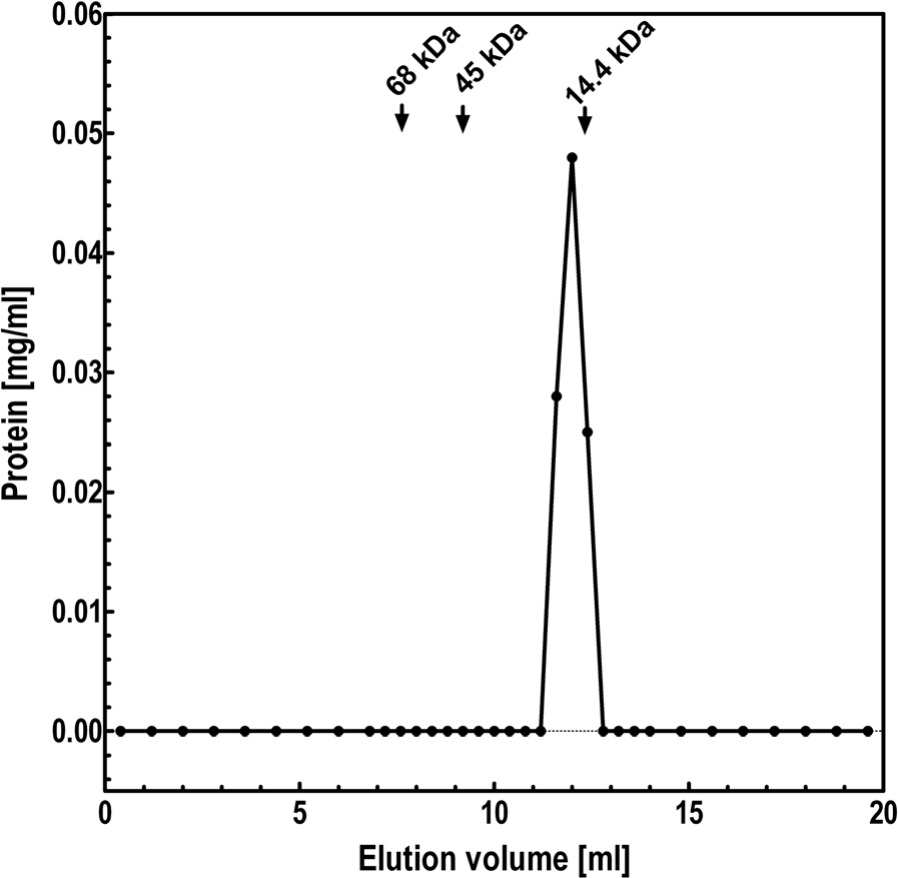
**Size exclusion HPLC analysis of SSB**_**BA**_**.** **Size exclusion HPLC analysis of SSB**_**BA**_**.** The native molecular mass of SSB_BA_ Fraction V was investigated using a TSK GS3000SW gel filtration column using A-100 as running buffer. Twenty micrograms of SSB_BA_ was injected and the column was eluted at 0.4 ml/min. and 0.4 ml fractions were collected. The gel filtration standards were BSA (68 kDa) and ovalbumin (44 kDa) and lysozyme (15 kDa).

Recombinant SSB_BA_ was highly soluble when expressed in *E. coli*. It was purified using a combination of ammonium sulfate fractionation as well as conventional ion exchange chromatography. These steps resulted in homogenous SSB_BA_ (Figure 2A). In SE-HPLC analysis of purified SSB_BA_ (100 μg/ml) the protein eluted as a single peak with an elution volume consistent with that of a monomer (Figure 3).

To test the biological activity of SSB_BA_ we measured its ability to stimulate its cognate DnaB, DnaB_BA_, using a FRET based DNA helicase assay. It was based on the ability of SSB_BA_ to stimulate DNA unwinding activity of its cognate DNA helicase, DnaB_BA_ (Figure 2). In the absence of SSB_BA_, DNA unwinding by DnaB_BA_ was limited (Figure 2B) and was greatly stimulated in the presence of SSB_BA_ (Figure 2C). The stimulation of the DNA helicase activity of DnaB_BA_ in the presence of the purified SSB_BA_ was >10 fold which was as expected for the cognate SSB [13].

### Mechanism of ssDNA binding by SSB_BA_

**Figure 4:**
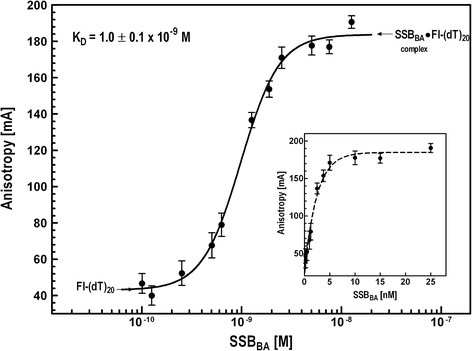
**Fluorescence anisotropy analysis of equilibrium ssDNA binding by SSB**_**BA**_**.** ssDNA binding was measured using fluorescence anisotropy of Fl-(dT)_20_ oligonucleotide probe using the fluorescence of its 5′-fluorescein moiety using 480 nm excitation and 540 nm emission of fluorescein as described in Materials & Methods. Titration was carried out with SSB_BA_ and fluorescence anisotropy of Fl-(dT)_20_ was measured. As indicated, anisotropy of free Fl-(dT)_20_ oligonucleotide probe was 44 ± 5 mA and that of the SSB_BA_·Fl-(dT)_20_ complex was 182 ± 6 mA. Anisotropy values were plotted against log of SSB_BA_ concentration and the plots were analyzed by nonlinear regression using Prism 6.0. The error bars indicate standard deviation. [Inset] A simple plot of the data fitted to single association hyperbolic function.

Fluorescence anisotropy-based titration of a DNA-fluorophore by the DNA binding protein is a direct methodology for determining affinity of protein-DNA complexes. Following this approach, 5′-Fluorescein-labeled oligo(dT)_20_ (Fl(dT)_20_) was used as a fluorescence anisotropy probe for analyzing SSB_BA_ and ssDNA interactions. In order to determine the binding constant for SSB_BA_, the interaction of SSB_BA_ with 5′-flourescein labeled oligo(dT)_20_ was examined at 25°C and in buffer B containing 0.1 nM Fl-(dT)_20_, 25 mM KCl and 5 mM Mg^+2^. Fl-(dT)_20_ was titrated with SSB_BA_ until saturation in anisotropy was observed. The anisotropy values at various SSB_BA_ concentrations were used to create a binding isotherm as a semi-log plot as shown in Figure 4. At very low SSB_BA_ concentrations, very small anisotropy changes and a flat plateau (~43 mA) were observed that were attributed to Fl-(dT)_20_. Upon further addition of SSB_BA_, the anisotropy value increased with an increase in SSB_BA_·Fl-(dT)_20_ complex formation. A sigmoid binding isotherm with saturation binding at high SSB_BA_ concentration was observed with maximum anisotropy of 184 ± 5 mA. At higher SSB concentration (≥ 1 μM), anisotropy did not change significantly (data not shown). Nonlinear regression analysis, using a sigmoidal dose–response equation, of the data allowed for determination of the SSB_BA_ concentration at which 50% of the ligand was in bound form (EC_50_); that value corresponds to the apparent dissociation constant. The K_D_ for SSB_BA_·Fl-(dT)_20_ complex was 1.0 ± 0.1 x 10^−9^ M. The Hill coefficient was 1.6 ± 0.6 indicating a possible binding of one to two molecules of SSB_BA_ to Fl-(dT)_20_, which is not surprising considering the fact that SSB_BA_ does not form a stable tetramer. A simple hyperbolic fit of the anisotropy data was also carried out (Figure 4 inset). The estimated K_D_ value was 1.2 ± 0.8 x 10^−9^ M. In order to determine the correct method of analysis of binding data, we have examined the “Goodness of Fit” using both models. The R^2^ value for the sigmoidal dose–response model was found to be 0.98, whereas, for the simple hyperbolic model was 0.94. The F-test using the values of degrees-of-freedom and absolute sum of squares, gave an F-value of 22.4 which correlated well with the R^2^ values and clearly demonstrated that the sigmoidal dose–response model as the correct model/equation for fitting anisotropy binding data for such analysis.

### Thermodynamics of ssDNA binding

**Figure 5:**
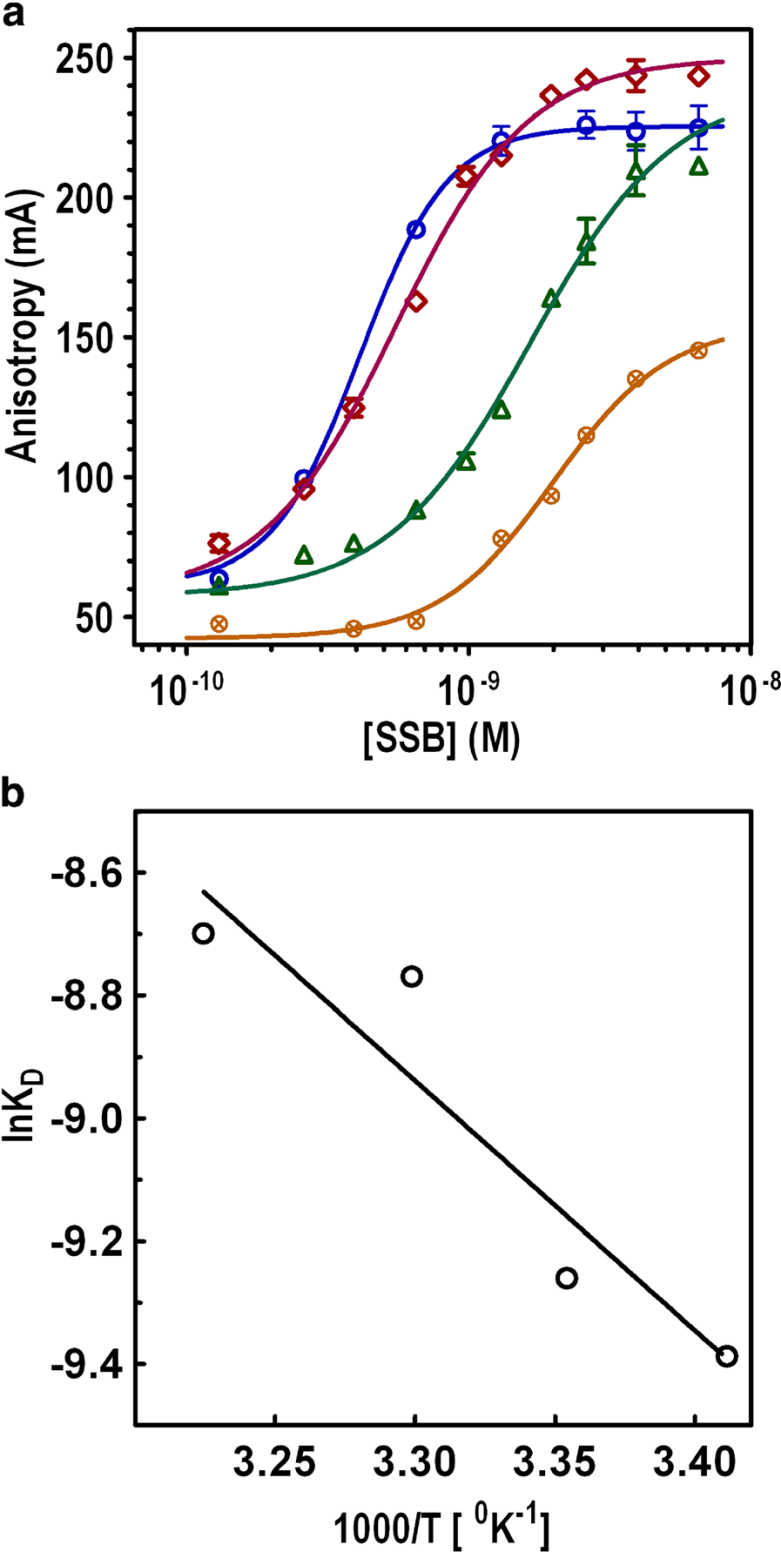
**Temperature dependence of SSB**_**BA**_**·ssDNA complex formation.** Temperature dependence of ssDNA binding by SSB_BA_ was measured at temperatures as indicated. **(a)** Binding isotherms for SSB_BA_ binding to ssDNA at seven temperatures: 20°C, 25°C, 32°C and 37°C are shown. The concentration of the oligonucleotide was 1.0 nM and 25 mM NaCl was added. **(b)** Van’t Hoff plot was made using the analysis of the K_D_ values obtained from the nonlinear regression of plot in 5A.

In order to understand the thermodynamics of SSB_BA_·ssDNA binding interactions, we have analyzed SSB_BA_ binding to ssDNA at different temperatures over a range of 20–37°C. The temperature-dependent binding isotherms for SSB_BA_ and Fl-(dT)_20_ are presented in Figure 5A. Higher anisotropy values observed at 20 or 25°C were due to glycerol used in this assay. As the temperature increased, an overall decrease in anisotropy value was observed for both the free and bound oligonucleotides. This overall decrease in anisotropy value is attributed to the decrease in viscosity of the solution with increasing temperature which was somewhat attenuated by adding glycerol. With a decrease in temperature, the viscosity as well as the anisotropy values appeared to increase [40]. Analysis of the binding curve data for K_D_ showed the dissociation constants increased steadily from 20°c to 37°C. We did not observe any significant change in the Hill coefficient with temperature. This increase in K_D_ could likely be due to the dissociation of the protein·DNA complex at higher temperatures. The DnaA_BA_·DNA complex was most stable at 20–25°C.

The dissociation constants obtained at varying temperatures were used to evaluate the thermodynamic properties of DNA binding. We have plotted the dissociation constants using the Van’t Hoff equation, lnK_D_ = −ΔH°/RT, where ΔH° is the enthalpy change and T and R are the temperature and gas constant respectively, with the dissociation constants derived from 20, 25, 30, and 37°C (Figure 5B). The plot is linear for temperatures 20°C to 37°C and it diverges from linearity below 20°C. The slope of the Van’t Hoff plot was used to derive the change in enthalpy (ΔH°) at 25°C (33.9 kJ mol^−1^). The change in entropy (ΔS°) was calculated to be 56.9 J mol^−1^ K^−1^. Thus, it appears that the formation of the SSB_BA_·ssDNA complex has a strong entropic or hydrophobic component to the overall protein-DNA interaction.

**Figure 6:**
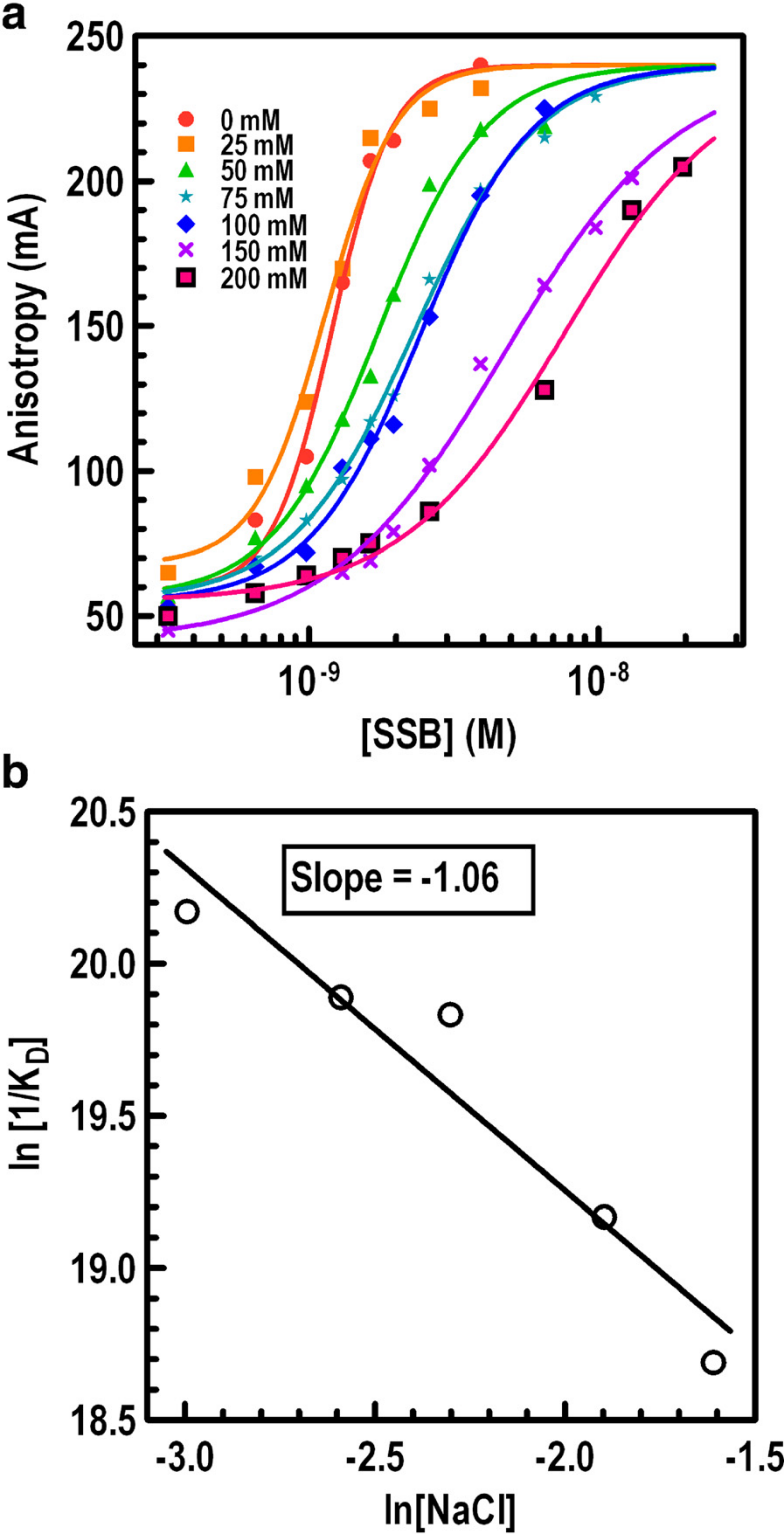
**Ionic strength dependence of SSB**_**BA**_**·ssDNA complex formation.** Ionic strength dependence of ssDNA binding by SSB_BA_ was measured at NaCl concentration as indicated. **(a)** Binding isotherms for SSB_BA_ binding to ssDNA at six NaCl concentrations: 0, 25, 50, 100, 150, and 200 mM. **(b)** Thermodynamic linkage plot for SSB_BA_ binding to ssDNA as a function of NaCl concentration. The net average number of ions released upon complex formation was derived from the slope of the plot.

In addition to hydrophobic interaction between SSB_BA_ and ssDNA, we analyzed the contribution of ionic interactions, if any, in the complex formation. Fluorescence anisotropy was used determine the K_D_ of formation of SSB_BA_·ssDNA complex as a function of NaCl concentration. The binding isotherm at different salt concentrations was generated using SSB_BA_ titration of ssDNA at 25°C (Figure 6A). Each titration curve fits according to a single site binding isotherm. The data shows that highest affinity binding occurs at 0–25 mM NaCl. The dissociation constant increased steadily from 0–250 mM NaCl. The most striking change was in the values of the Hill coefficient. At 0 mM NaCl, it was 1.9 ± 0.6 and steadily decreased to 0.9 ± 0.1 at 150 mM NaCl and beyond. It could indicate that at 0 mM NaCl, SSB_BA_ was forming a predominantly dimeric structure which transformed into monomeric at higher NaCl concentration. However, at very low ionic strength, non-specific protein-protein interactions could not be ruled out. A thermodynamic linkage plot for ssDNA binding as a function of NaCl (Figure 6B) was generated from the K_D_ values obtained from Figure 6A. The presence of a negative slope was indicative of a net ion release [41]. The data were analyzed using the following equation to determine the number of ions released upon binding:

(1) $$\Delta {\text{n}}_{\text{ions}}=1\text{n}\left(1/{\text{K}}_{\text{D}}\right)/1\text{n}\left[\text{NaCl}\right]$$

The analysis suggests that upon SSB_BA_·ssDNA complex formation, only one ion was released from the protein-DNA interface. These results appeared to indicate a small but significant contribution of ionic interaction in the ssDNA binding.

### Structural analysis of SSB_BA_ by homology modeling

The SSB_BA_ sequence was further analyzed for secondary structure using Rosetta software (http://robetta.org/fragmentsubmit.jsp). Rosetta analysis indicated that there are at least five significant β strand structures and a single α-helix in the N-terminal half of the molecule (data not shown). The structure between the residues 101–170 appeared to be a random coil. These secondary structures are consistent with known features of SSB_EC_ monomer, as determined earlier by X-ray crystallography [37,39,42].

**Figure 7:**
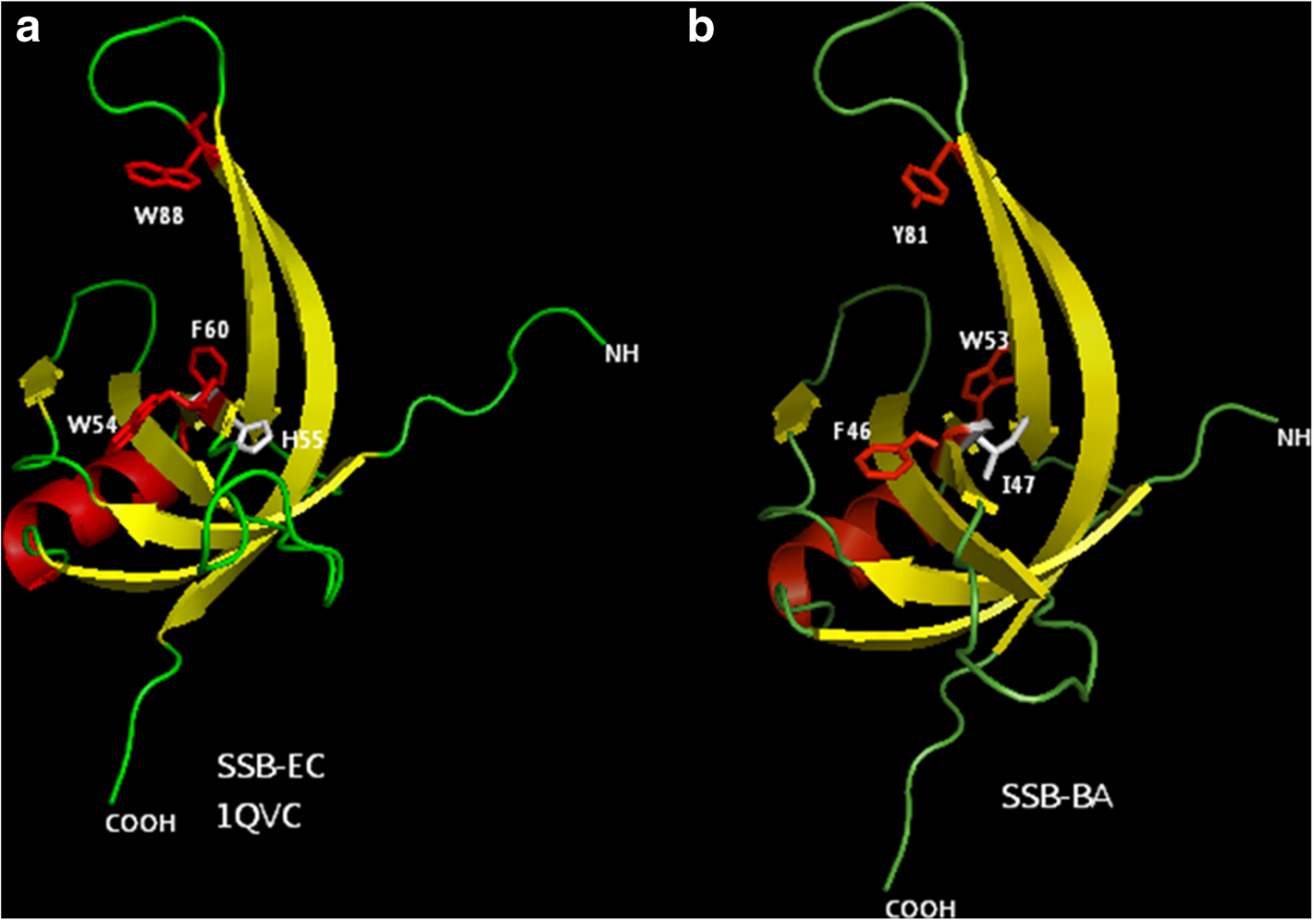
**Homology based modeling of SSB**_**BA**_**. (a)** Model of the SSB_EC_ derived from the crystal structure of a chymotrypsin truncated SSB_EC_ monomer lacking 42 C-terminal residues (PDB ID 1QVC). **(b)** Model of the SSB_BA_ derived from the crystal structure SSB_EC_ monomer (PDB ID 1QVC). Both of these structures were generated using PyMol. In SSB_EC_, Trp55, Trp89, and Phe61 are important in the ssDNA binding, which are replaced by Phe47, Tyr82, and Trp53 respectively in SSB_BA_. Most notably, His56 residue of SSB_EC_, required for monomer-monomer interaction is replaced by Ile48 in SSB_BA_[39].

Alignment of SSB_BA_ sequence with sequences of other prokaryotic SSBs did not provide any clue to the basis of its ssDNA binding properties (Figure 1). Therefore, the three-dimensional structure of SSB_EC_ derived from X-ray crystallography [37] was explored. The significant sequence homology of SSB_BA_ with *E. coli* SSB allowed us to develop a putative three dimensional model of SSB_BA_ using homology based modeling. The initial modeling was done using the SWISS-MODEL server [43,44]. Further refinement of the model by energy minimization was carried out using SYBYL 8.1 (Tripos Inc., St. Louis, MO) molecular modeling software using the SSB crystal structure, PDB ID: 1QVC, as the structure template [37]. The structures for *E. coli* SSB_EC_, 1QVC and SSB_BA_ were visualized using PyMOL (The PyMOL Molecular Graphics System, Version 1.3, Schrödinger LLC). Structures of both SSBs are presented in PyMol in Figure 7.

SSB proteins are known to bind ssDNA through their oligonucleotide/oligosaccharide binding fold (OB fold) as described by Murzin [38]. The OB fold is characterized by a β-barrel consisting of five β-strands capped by an α-helix. Despite sequence differences between SSB_BA_ and SSB_EC_, the OB fold observed in SSB_EC_ remained intact in SSB_BA_ including the β-turn regions, particularly L45 between β sheets 4 and 5 (Figure 7). It has been shown that the β sheet 1 of SSB_EC_ with the sequence VNKVILV is in the monomer-interface of the SSB_EC_ dimer [39]. In SSB_BA_, this β sheet remains partially intact (NKVILV) with the loss of the Val5 residue. However, the His56 of one monomer in SSB_EC_ forms a hydrogen bond with Asn6 and the carbonyl oxygen of Leu83 of another monomer, which is essential for a stable dimer/tetramer formation. Although Asn6 (Asn2 in SSB_BA_) and Leu83 (Leu76 in SSB_BA_) remained conserved, one of the most important residues, His56 in SSB_EC_ was altered to Ile (Ile47) in SSB_BA_ (Figure 1). It should be noted that in the temperature-sensitive *E. coli* mutant, *ssb-1*, His56 was mutated to Tyr56 leading to the *ts*-phenotype. *E. coli ssb-1 ts-*mutant does not form a stable tetramer at non-permissive temperature [5,45]. Thus, the lack of this His residue in SSB_BA_ will likely hinder a stable dimer formation.

The SSB_EC_ tetramer is formed by the interaction of two dimers [37,39]. The dimer-dimer interface involves two six-stranded surfaces, each comprised of β1, β4, and β5 from two monomers. The structure of SSB_BA_, as shown in Figure 7, could form such a tetramer interface, had it not been for the difficulty associated with the dimer formation. It has been shown with a number of SSB crystal structures that a network of hydrogen bonds among the side chains in this six-stranded interface is necessary for a stable tetramer formation. The residues that were shown to be important in SSB_EC_ for this network of hydrogen bond formation are Lys8, Tyr79, Gln77, Glu81, and Gln111. Sequence comparison (Figure 1) between SSB_EC_ and SSB_BA_ indicated that all of these residues in SSB_BA_ underwent changes and are as follows: Lys8→Arg2, Tyr79→Gly71, Gln77→Leu69, Gln83→Arg75, and Gln111→Phe104. Although all of the changes may not be significant, three of these five changes are significant in terms of hydrogen bond formation. Therefore, these amino acid changes in SSB_BA_ are likely to impede tetramer formation further. Taken together, inhibition of both monomer-monomer interaction leading to dimer formation as well as dimer-dimer interaction leading to tetramer formation, the amino acid sequence of SSB_BA_ does not support formation of stable dimer or tetramer.

### Analysis of the structure of ssDNA binding pocket in SSB_BA_

Single-stranded DNA binding by prokaryotic SSBs has been shown by several groups to be carried out exclusively by tetrameric forms of SSBs containing four OB folds or dimeric forms with each monomer containing two OB folds [37,39,46]. Thus, the presence of four OB folds in SSBs appears essential for high affinity ssDNA binding. Our studies indicated that SSB_BA_ bound ssDNA with very high affinity (1.0 ± 0.1 x 10^−9^ M) even though it did not appear to form a stable tetramer in the absence of DNA at the concentration range examined (Figures 3 & 7).

**Figure 8:**
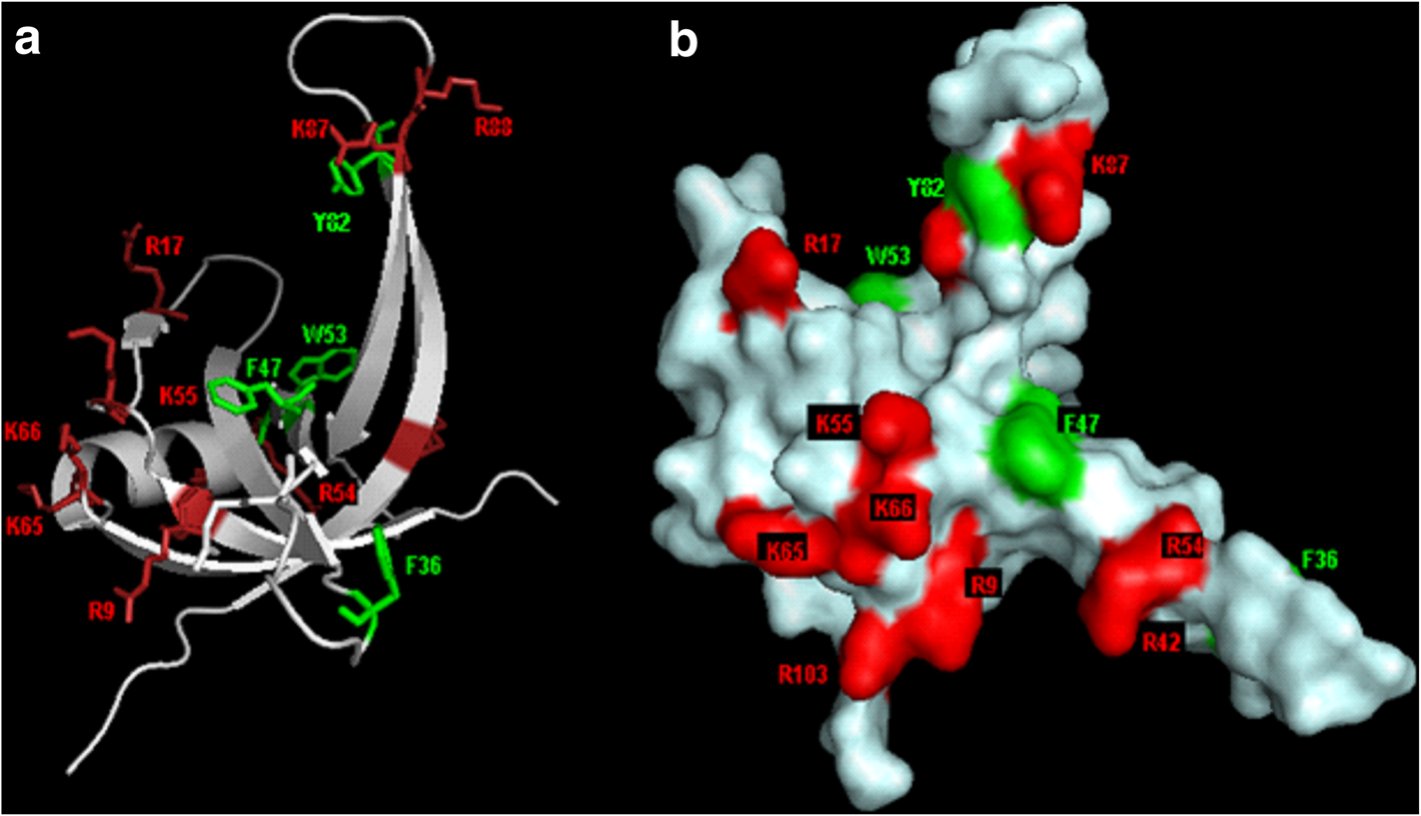
**Surface topology and charge distribution on the surface of SSB**_**BA**_**that are involved in ssDNA binding.** Hydrophobic (in green) and basic residues (in red) that are likely involved in ssDNA binding are depicted. **(a)** Hydrophobic residues (Phe36, Phe47, Trp53, and Tyr82) are depicted in green sticks and basic residues (Arg 9, 17, 54, 88 and Lys 55, 65, 66, 87) are presented in red sticks attached to the backbone. **(b)** Electrostatic surface potential of the SSB_BA_ are created from the structure in Figure 7A using the program PyMOL. Hydrophobic, positive, and neutral potential surface are displayed and colored green, red, and white, respectively.

The amino acid residues in SSB_BA_ that are homologous to the residues in other SSBs, particularly SSB_EC_, that are known from crystallographic studies to bind to ssDNA were analyzed. Several hydrophobic residues, Trp89, Trp55, and Phe61, in SSB_EC_ have been identified as involved in ssDNA binding through base stacking interactions [39]. In Helicobacter pylori SSB (SSB_HP_), Phe37, Phe50, Phe56, and Trp84 are involved in base stacking interactions with ssDNA [47]. These hydrophobic residues (Phe36, Phe43, Trp53, and Tyr81) with changes remained conserved in SSB_BA_ (Figure 1). As shown in Figure 8, these aromatic side chains are exposed in the DNA binding groove of the OB fold so that ssDNA bases could have stacking interactions. In addition, ssDNA binding to SSB requires a large number of positively charged residues for the formation of ionic bridges with the phosphodiester backbone of ssDNA. A large number of Arg and Lys residues were observed in and around the groove as shown in Figure 8. Among these, Arg9, Lys12, Arg17, Arg42, Arg54, Lys55, Lys65, Lys66, Lys87, Arg88, Arg103 appeared to be in close proximity of the DNA binding groove and form ionic bridges with the phosphodiester backbone. Consequently, the high affinity ssDNA binding observed with SSB_BA_ could be due to basic as well as aromatic residues in its DNA binding groove.

### Subunit structure of SSB_BA_ in the SSB_BA_·ssDNA complex

**Figure 9:**
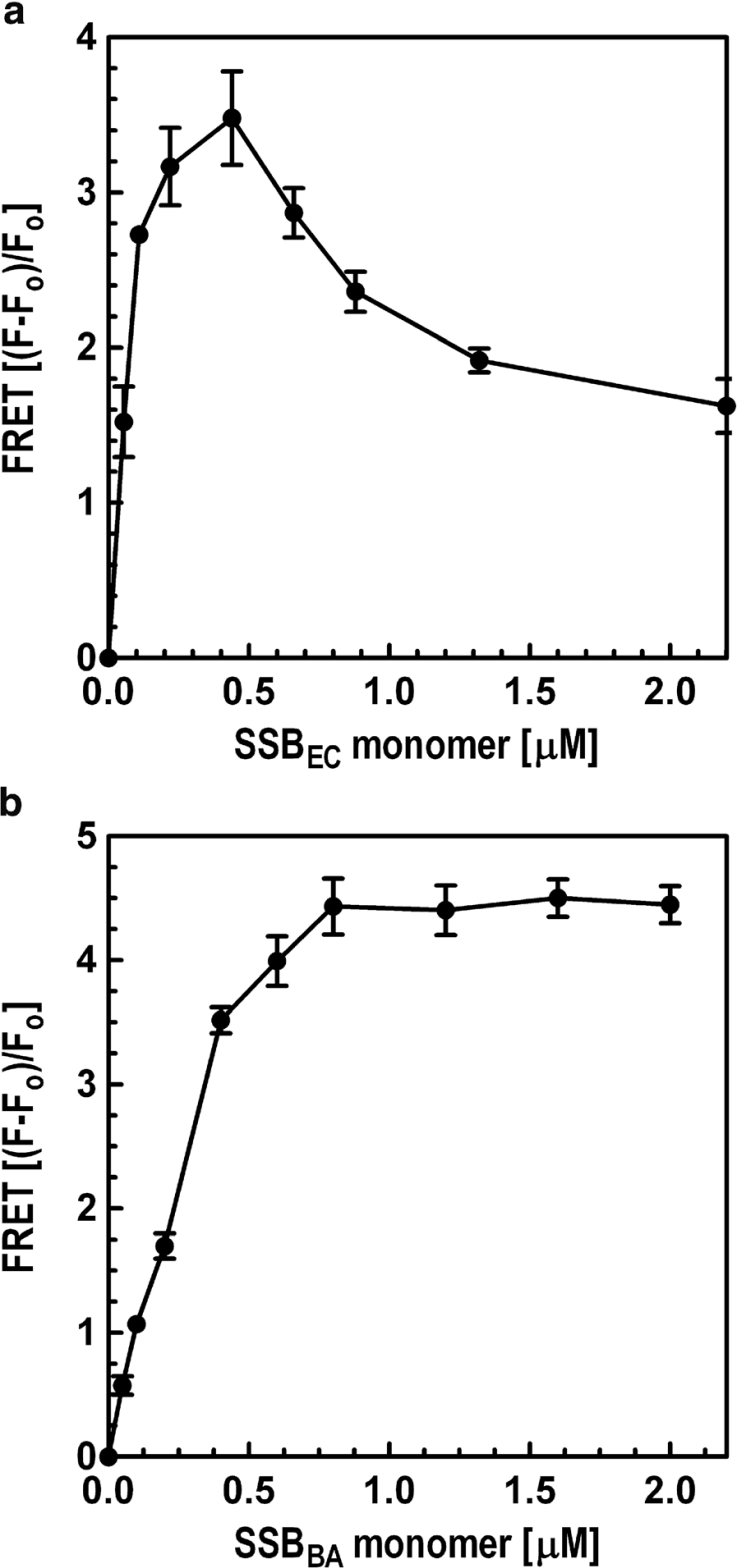
**Analysis of binding modes, (SSB)**_**35**_**and (SSB)**_**65**_**, of SSB**_**EC**_**and SSB**_**BA**_**by FRET analysis.** Protein-ssDNA binding and FRET assays were carried out as described in Materials & Methods [26]. The FRET substrate was 40 nM 5′-Cy5-(dT)_70-_Cy3-3′. **(a)** Analysis of SSB_EC_ binding to 5′-Cy5-(dT)_70-_Cy3-3′. Both (SSB)_65_ and (SSB)_35_ modes are clearly observed (22). **(b)** Analysis of SSB_BA_ binding to 5′-Cy5-(dT)_70-_Cy3-3′. Only (SSB)_65_, not (SSB)_35_, mode was observed.

Our studies demonstrated that SSB_BA_ was capable of high-affinity binding of ssDNA. Was it possible that the protein could form higher order structures, possibly tetramers, upon binding long ssDNA templates? Tetrameric *E. coli* SSB binds ssDNA in two distinguishable forms; SSB_35_ and SSB_65_[26,48]. The SSB_35_ form binds approximately 35 nt and the SSB_65_ form binds approximately 65 nt on a (dT)_70_ template. In the SSB_35_ form, only two subunits of the tetramer make contacts with the DNA, whereas, in SSB_65_ form all four subunits of the tetramer make contacts with the DNA. These two forms can be distinguished by fluorescence resonance energy transfer (FRET) using a long ssDNA labeled in each end with Cy3 and Cy5 fluorophores [26]. The SSB_65_ form produces high FRET and the SSB_35_ form produces attenuated FRET. This approach was utilized using a seventy nucleotide, (dT)_70_, oligonucleotide labeled with a 3′ Cy3 fluorophore and a 5′ Cy5 fluorophore (Cy5-(dT)_70_-Cy3) to test our hypothesis that SSB_BA_ forms higher order structures upon DNA binding. With this ssDNA substrate, we anticipated that SSB_BA_ in SSB_35_-mode would produce attenuated FRET and SSB_65_-mode would produce high FRET. Binding of SSB_EC_ to Cy5-(dT)_70_-Cy3 oligonucleotide was first analyzed to test the validity of our assay. The results are presented in Figure 9. FRET was measured using 515 nm excitation wavelength and 665 nm emission wavelength. The emission intensity was corrected for Cy5 contribution to 665 nm emission. FRET was defined by (F-F_0_)/F_0_ where F_0_ and F are the corrected 665 nm emission intensities of the 200 nM 5′-Cy5(dT)_70_Cy3-3′ oligonucleotide in the absence and presence of SSB respectively. In the titration with SSB_EC_, sharp increase in FRET with initial titration with SSB_EC_ reaching a peak at 0.44 μM concentration was seen. Upon further titration with SSB_EC_, the FRET decreased substantially and reached a plateau at concentrations higher than 1.1 μM. These results with SSB_EC_ are comparable to that observed by Roy et al. [26]. However, our experiments required a somewhat higher concentration of SSB_EC_ than reported by Roy et al. [26], which could be due to different buffer systems and ssDNA concentration. Thus, at 0.44 μM SSB_EC,_ the high FRET (SSB_EC_)_4_-(dT)_70_ complex was observed and at or above 1.1 μM SSB_EC_, intermediate FRET (SSB_EC_)_8_-(dT)_70_ complex was seen.

**Figure 10:**
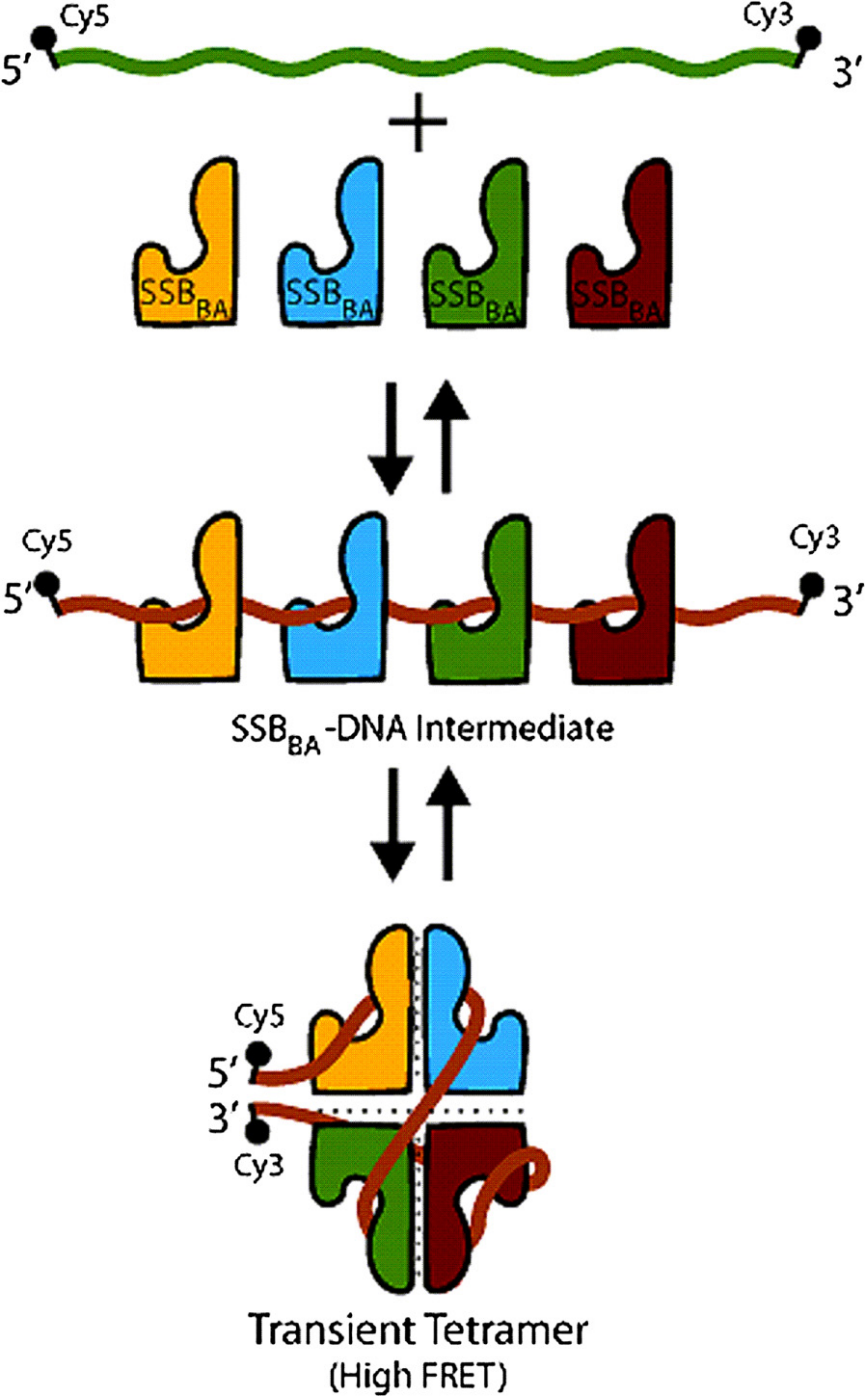
**A hypothetical model for the formation of SSB**_**BA**_**tetramer upon ssDNA binding.** The cartoon depicts a hypothetical model for the formation of ssDNA·(SSB_BA_)_4_ complex with a wrapped ssDNA that is bound to all four SSB_BA_ monomers in SSB_65_ mode.

Next, SSB_BA_ binding to Cy5(dT)_70_Cy3 oligonucleotide was analyzed. The Cy5(dT)_70_Cy3 oligonucleotide was titrated with SSB_BA_ as described above for SSB_EC_. Similar to SSB_EC_, FRET increased linearly with increasing concentration of SSB_BA_, and reached a plateau at 0.8 μM Surprisingly, only a high FRET form of (SSB_BA_)-(dT)_70_ complex was observed. However, initial slope of the plot is very different from that observed with SSB_EC_ which is a stable tetramer. Therefore, it appeared that the high FRET form of the complex was formed but required higher concentration of SSB_BA_. The high FRET form of (SSB_EC_)-(dT)_70_ complex is a tetrameric complex in which all four monomers bind ssDNA. The high FRET form of (SSB_BA_)-(dT)_70_ complex should have the same oligomeric structure as the high FRET form of (SSB_EC_)-(dT)_70_ complex. As SSB_BA_ lacked stable tetramer formation, it required higher (~two fold) SSB concentration to form the high FRET complex. The reason is that this complex is not formed by a single binding event as is the case with SSB_EC_ complex. It involves four binding and one structural rearrangement steps. As individual monomers are binding, there are four separate binding constants (K1, K2, K3, and K4) involved in the (SSB_BA_)_4_-(dT)_70_ complex formation. It will be erroneous to assume that they are all 1 nM. Certainly K1 is 1 nM as observed in Figure 4. Other binding constants (K2, K3, and K4) are likely to be higher than 1 nM due to steric hindrance which is particularly important for K4 involving the binding of the fourth monomer. The (dT)_70_ is small and can accommodate only four SSB_BA_ resulting in progressive lack of sufficient open DNA for second, third and fourth SSB_BA_ monomers to bind. Therefore, it is very likely that K2 and K3 are higher and K4 is substantially higher than 1 nM which explains a higher SSB_BA_ concentration to achieve a high FRET complex. Based on this reasoning of the (SSB_BA_)_4_-(dT)_70_ complex formation, as described above, our data actually supports the model in Figure 10. Lack of observation of an intermediate FRET form of the (SSB_BA_)-(dT)_70_ complex in this study indicated that a (SSB_BA_)_8_-(dT)_70_ complex probably did not form in appreciable amount even with high proportional levels of SSB_BA_. Based on these reasonings of the (SSB_BA_)_4_-(dT)_70_ complex formation, as described above, our data led to the proposal of the model presented in Figure 10 for the (SSB_BA_)_8_-(dT)_70_ complex.

## Discussion

SSB protein is required for a variety of processes such as DNA replication, recombination and DNA repair despite its lack of any enzymatic activity [5,9,49]. Among its multifaceted cellular activities, a common feature of all of these processes is to bind ssDNA with high affinity and protect it from reannealing and/or degradation. Of emerging importance is SSB’s role in protein-protein interaction during various DNA transactions. Most studies involving SSB proteins demonstrated that ssDNA wraps around a tetrameric form of SSB.

### SSB_BA_ does not form a stable tetramer

*E. coli* SSB_EC_ is a stable tetramer with high solubility and tremendous thermal stability [1]. The majority of prokaryotic cellular SSBs are homotetramers, where each monomer harbors an OB fold. However there are exceptions. SSB_DR_ from *Deinococcus radiodurans* is a homodimer, where each monomer is quite large and contains two OB folds [37-39,46]. Each OB fold is capable of binding ssDNA independently. In both cases, a stable SSB protein complex with four OB folds is required for ssDNA binding. SSB_BA_ was found to be not tetrameric at or above ambient temperature by size exclusion HPLC (Figure 3). This physicochemical property of SSB_BA_ is closely comparable to the T4 bacteriophage SSB, which is monomeric.

### Molecular basis of SSB_BA_ structure

Sequence alignment and three dimensional structure of SSB_BA_, generated by homology-based modeling were utilized to probe the molecular basis of ssDNA binding (Figures 1). Sequence alignment and secondary structure prediction (data not shown) clearly indicated the presence of an OB fold in SSB_BA_, which is a characteristic of SSBs and required for high affinity ssDNA binding (Figure 4).

Both sequence alignment and homology modeling (Figures 1 & 10) indicated lack of several residues that are important for monomer-monomer and dimer-dimer interactions leading to the formation of a stable tetramer in SSB_EC_. His56 (as well as Glu54) of β sheet 3 (Figure 1) in SSB_EC_ plays an important role by forming a hydrogen bond with Asn7 in β sheet 1, the carbonyl group in Leu84 and Thr100 at the base of loop L45. Notably, in *E. coli* temperature-sensitive mutant, *ssb-1*, His56 is mutated to Tyr56 [5]. This mutant does not form a stable tetramer with respect to monomers at non-permissive temperatures [5,45,50]. Thus, a lack of the corresponding His residue (His→Ile change) in SSB_BA_ may be one of the important contributors to its structure. Moreover, in *B. anthracis* the sequence Glu54. Trp55.His56 in the *E. coli* β sheet 3 is altered to Asp46.Phe47.Ile48 (Figure 1). This change did not alter the β sheet structure but may have altered the contributions of these residues in the monomer-monomer interaction in the stable dimer and tetramer formation. It appears that although β sheet 1 remained conserved in SSB_BA,_ this region lacks the valine residue which may have attenuated the interaction of the β sheet 1 with β sheet 1′ in the monomer-monomer interface of the dimer. The shortening of the N-terminus in SSB_BA_ may also have deleterious effect in the interactions involving H-bonds in this region and can contribute to the lack of tetramer formation. Taken together, the amino acid residue substitutions in SSB_BA_, as described above, are likely contributed to the disruption of monomer-monomer interaction leading to dimer formation.

The dimer-dimer interface in the SSB_EC_ tetramer is primarily a six-stranded β sheet-mediated. Residues that are important in SSB_EC_ for the network of hydrogen bond formation at the dimer-dimer interface are Lys8, Tyr71, Gln77, Glu81, and Gln111 [39]. Sequence comparison (Figure 1) between SSB_EC_ and SSB_BA_ indicated that all of these residues in SSB_BA_ underwent alteration and are as follows: Lys8→Arg3, Tyr79→Gly71, Gln77→Leu69, Gln83→Arg75, and Gln111→Phe104. Some of these changes are chemically significant leading to possible disruption of the network of hydrogen bond formation that is required for a stable tetramer formation. In addition, Gln77 and Gln111 are located in the dimer-dimer interface and have been implicated in the tetramer formation. As described earlier, Gln111 is altered to Phe104 in SSB_BA_. An equally significant change is observed with Gln77 which is changed to Leu69 in SSB_BA_. Taken together, these changes in amino acid sequence may disrupt both monomer-monomer and dimer-dimer interactions leading to a monomeric SSB_BA_ at a physiological temperature.

### Energetics of SSB_BA_·ssDNA binding

Protein-DNA recognition and binding involve complex interactions. Earlier, we have used fluorescence anisotropy analysis of DNA binding by *E. coli* DNA primase and determined the thermodynamic parameters of protein-DNA interaction (38). We have used a similar analysis to probe the ssDNA binding by SSB_BA_. In addition, we have analyzed contribution of electrostatic and ionic interactions in the binding by analyzing the dependence of binding on the ionic strength of the environment. Together, these two analyses provided a detailed picture of the forces in SSB_BA_·ssDNA binding.

The K_D_ values were determined at different temperatures (20–37°C) (Figure 5). Our data showed that although SSB_BA_ was able to bind DNA at a wide range of temperatures, it bound with the highest affinity at 20–25°C. The free energy change for SSB·ssDNA association was −23 kJ mol^−1^ at 25°C. Using the two equations: ΔG° = −RT lnK_D_ and ΔG° = ΔH°−TΔS° and the slope of the plot, we determined that ΔS° was ~188 J mol^−1^ K^−1^ in this temperature range.

We determined the K_D_ value of SSB_BA_ binding to ssDNA at different salt concentrations (0–200 mM NaCl) (Figure 6A). The binding is progressively weakened with an increase in ionic strength. The K_D_ values were then analyzed using a linkage plot to determined ionic interactions in the binding. The negative slope of the plot in Figure 5B indicated a release of ions from. Our analysis determined the release of one Na^+^ and one Cl^-^ ion during the binding process. In addition, our results also pointed out that SSB_BA_ likely formed a tetrameric species at 0 mM NaCl and became monomeric at higher NaCl concentration. It is perhaps possible that the tetramer formation could be dependent on the ionic strength.

Thus our results suggest that ionic interaction or salt bridge formation between the protein and the DNA made specific contribution to the overall free energy change. In order to determine the contribution we first extrapolated K_D_ value of the complex at infinite salt concentration (K_D_^∞^) by nonlinear regression of K_D_ versus log[NaCl] plot (data not shown). The value of ΔG°_ionic_ was −8 kJ mol^−1^.

### Mechanisms of ssDNA binding by SSB_BA_

Despite differences between its Gram-negative counterpart, SSB_BA_ bound to ssDNA with high affinity (Figure 4). The ssDNA binding affinity (K_D_) for a SSB_BA_ monomer binding to a small oligonucleotide was 1.0 ± 0.1 x 10^−9^ M at 25°C. Even though many changes in amino acid sequence of SSB_BA_ directly relate to ssDNA binding, such as Trp55→Phe47, Trp90→Tyr81, Phe61→Trp53, the changes are not drastic enough to alter ssDNA binding (Figure 8). Three dimensional structure as well as electrostatic surface potential in Figure 8 indicates that ssDNA binding remained unperturbed. A temperature-sensitive mutant of *E. coli* SSB, *ssb-1*, is unable to form a stable tetramer at a non-permissive temperature [5,45,50]. This mutant is also defective in supporting DNA replication at non-permissive temperature. Thus, it appears a SSB tetramer formation is a prerequisite for DNA replication. Consequently, we sought to explore whether ssDNA template could influence the ability of SSB_BA_ to form tetramers upon DNA binding. A likely possibility is that SSB_BA_ is capable of forming a normal tetrameric structure containing four OB-folds, as seen in other SSBs, upon sufficiently long ssDNA. This possibility was examined using a recently developed FRET assay for SSB·ssDNA interaction [26].

Previous studies with SSB_EC_ have shown that its SSB_35_ and SSB_65_ binding modes can be distinguished by a FRET assay [26]. Both of these ssDNA binding modes require a tetrameric (or di-tetrameric) structure of bound SSB. A similar FRET assay was used to probe the structure of SSB_BA_ in ssDNA bound state. As the ssDNA binding constant of SSB_BA_ is very high, the possibility that it may form a tetramer only in the ssDNA bound state was investigated. As shown in Figure 9A, SSB_EC_ formed both SSB_35_ and SSB_65_ structures with the 5′-Cy5(dT)_70_Cy3-3′ oligonucleotide as evidenced by FRET analysis. As described earlier, SSB_35_ represented the intermediate-FRET complex and SSB_65_ represented the high-FRET SSB-ssDNA complex. At a low SSB to dT_70_ ratio, it formed the high FRET complex and at a high SSB to dT_70_ ratio, it formed the intermediate FRET complex. In the FRET analysis, SSB_BA_ formed only a high-FRET complex but not the intermediate-FRET complex (Figure 9B). In addition, the slope of the plot with SSB_BA_ is different from that of SSB_EC_. As SSB_EC_ is a stable tetramer, the high FRET complex formed rapidly with increasing SSB concentration and it formed much slowly with SSB_BA_ because of the lack of a stable tetramer formation. Our results appeared to indicate (i) a tetrameric structure of SSB_BA_ in the SSB_BA_-ssDNA complex, and (ii) the SSB_BA_-ssDNA complex was formed only in the SSB_65_ mode. Due to high affinity of ssDNA binding, perhaps four monomers can bind the oligo(dT)_70_ prior to the tetramer formation. Once this multi-SSB_BA_ complex is formed, the bound SSB_BA_ monomers undergo conformational transition and form tetrameric structure in SSB_65_ mode. A hypothetical model is proposed in Figure 10. In this proposed model, all four monomers in the tetramer would first bind to the ssDNA, which would likely lead to the formation of the SSB_65_ complex and prevent the formation of a SSB_35_ complex. In the case of SSB_BA_, a two-fold higher concentration of protein was needed to observe the high-FRET complex. We believe this is due to the following reasons. First, the SSB_BA_ is in essence a mutant form of SSB_EC_ and as a result its ssDNA binding mechanism is likely somewhat different. Second, higher concentration of SSB_BA_ might have favored the binding of all four monomers to the ssDNA. Initial slope of the plot in Figure 9B appeared to support this pathway.

SSB_BA_ appears to undergo structural transformations which may support its high affinity binding to ssDNA. Its structure is due to a cumulative effect of multiple changes in key amino acid residues in its sequence which resulted in the loss of stable tetramer formation. Nonetheless, the SSB_BA_ bound oligo(dT)_20_ with high affinity as shown in Figures 4, 5, 6. Therefore, multiple monomers will bind to oligo(dT)_70_ due to its long size. Thus, it is reasonable to assume that four monomers are binding to a long ssDNA (≥70 nucleotides). FRET data presented in Figure 9 established that SSB_65_-like structure is being formed upon oligo(dT)_70_ binding. Therefore, the ssDNA binding is leading to the formation of an SSB_65_ complex in which ssDNA is bound to a SSB_EC_-like tetrameric structure. We have proposed a hypothetical model, presented in Figure 10, which may explain the mechanism of formation of a SSB_BA_ tetramer assembly upon ssDNA binding which require further studies of such complex formation. The proposed model represents a stepwise process by which SSB_BA_ can achieve high affinity DNA binding through a tetramer formation. This mechanism of SSB-ssDNA complex formation and its reversal may aid in the rapid removal of SSB, a necessary step, by enzymes such as a DNA polymerase during DNA replication as well as in other processes. In essence, SSB_BA_ could actually be more effective than its tetrameric orthologs in executing its multifaceted functions in cellular DNA transactions.

## Conclusions

Our studies suggest that the structural properties of SSB_BA_ differ from that of its Gram-negative counterpart, SSB_EC_, and that furthermore its structure is modulated in the presence an ssDNA template. It is noteworthy that despite complexities in structure and oligomerization, SSB_BA_ retains high-affinity ssDNA binding, which is its primary function. Its unique structure may be due to the cumulative effect of multiple key amino acid changes in its sequence during evolution, leading to alteration of stable dimer and tetramer formation. In the presence of a long ssDNA (≥70 nucleotides) appears to form with SSB_BA_ a SSB_65_ complex in which ssDNA is bound to all four SSB monomers in a tetrameric structure. A proposed model may explain the mechanism of such SSB_BA_-ssDNA complex formation through a transient tetramer formation. This model indicates that SSB_BA_ may be more efficient in assembly and disassembly of the protein-DNA complex particularly during DNA replication. The physiological consequence(s) of the unusual structural dynamics of SSB_BA_, could be significant. Further studies are required to fully elucidate the role of protein·DNA and protein·protein interactions on SSB_BA_ protein structure.

## Methods

### Nucleic acids and other reagents

Ultra pure nucleotides were obtained from GE Biosciences (Piscataway, NJ) and were used without further purification. All other chemicals used to prepare buffers and solutions were reagent grade and were purchased from the Fisher Chemical Company (Pittsburgh, PA). HPLC ion exchange columns, ion exchange chromatography matrix, and the Bio-Cad 20 HPLC instrument were from Applied Biosystems Inc., Woburn, MA. The gel filtration column, TSK gel 3000SW, was from Tosoh Bioscience, King of Prussia, PA. Custom oligonucleotides for PCR and fluorescently labeled oligonucleotides were from Sigma-Aldrich (St. Louis, MO).

### Buffers

Lysis buffer contained 25 mM Tris–HCl, (pH 7.9), 10% sucrose, 250 mM NaCl, and 0.001% NP40. Buffer A contained 25 mM Tris–HCl (pH 7.5), 5 mM MgCl_2_, 10% glycerol, 5 mM DTT, and NaCl in mM as indicated in the subscript. Buffer B, used for all fluorescence studies, contained 20 mM Hepes-NaOH (pH 7.5), 5 mM MgCl_2_ and 1 mM DTT and 25 mM (unless otherwise indicated) ultrapure NaCl. In temperature and salt titration experiments, buffer B containing 5% ultrapure glycerol was used. Buffers for fluorescence measurements were prepared with HPLC-grade water (with minimal background fluorescence), fluorescence grade reagents, and filtered through a 0.2 μm nylon filter, examined for background fluorescence and Raman spectrum before use in anisotropy measurements. Background fluorescence was subtracted where necessary.

### Cloning and expression of SSB_BA_

The SSB_BA_ gene was amplified by PCR using *B. anthracis* genomic DNA, obtained as a gift from Dr. Theresa M. Koehler of the University of Texas Houston Health Science Center, Houston (33, 34). This ORF codes for a 172 amino acid polypeptide with a predicted molecular weight of 19.2 kDa. The amplified gene was cloned into a pET29b vector (Novagen, Inc., Madison, WI) under the control of a T7 promoter (pET29b-SSB_BA_ recombinant plasmid). The presence of the correct insert was confirmed by DNA sequencing. The SSB_BA_ protein was over-expressed in *E. coli* strain BL21(DE3)RIL (Agilent Technologies Inc., Santa Clara, CA) harboring pET29b-SSB_BA_ plasmid. Cells harboring the recombinant plasmid were grown in 2X-YT media containing 50 μg/ml of kanamycin, 20 μg/ml of tetracycline and 12 μg/ml of chloramphenicol with shaking at 37°C to an optical density at 600 nm of 0.4. IPTG (isopropyl-β-D-thiogalactopyranoside) was added to a final concentration of 0.25 mM. The cells were shaken for an additional two hours at 25°C, then harvested by centrifugation for 10 min at 5,000 x *g*. The cells were resuspended in 2.5% of the original culture volume of lysis buffer at 4°C and stored at −80°C until further use.

### Purification of SSB_BA_

Cells were thawed, adjusted to pH 8.0 with 1 M Tris base, and lysed using 0.25 mg/ml lysozyme, 5 mM MgCl_2_, 5 mM spermidine·HCl, and 2.5 mM DTT via incubation at ambient temperature for 60 min. The mixture was Dounce homogenized followed by centrifugation. The lysate was centrifuged at 43,000 x g for 30 min at 23°C. The supernatant was precipitated overnight using 0.25 g/ml ammonium sulfate at 4°C. This precipitate was collected by centrifugation at 43,000 x g for 30 min at 4°C, and dissolved in buffer A_0_ (Fraction II). Fraction II was clarified by centrifugation at 43000 x g for 30 min. All steps were carried out at ambient temperature unless otherwise indicated.

The salt concentration of Fraction II was adjusted to the conductivity of buffer A_50_ by diluting with buffer A_0_. The protein fraction was then passed through a 5 ml POROS-Q column equilibrated with buffer A_50_. SSB_BA_ protein was eluted with a 150 ml gradient from A_100_ to A_500_. The SSB_BA_ fractions, identified by SDSPAGE, were pooled (fraction III). The salt concentration of Fraction III was adjusted to the conductivity of buffer A_50_ by diluting with buffer A_0_. Diluted Fraction III was bound to a 5 ml S-Sepharose column equilibrated with A_50_. SSB_BA_ was eluted using a 150 ml gradient from A_100_ to A_500_. Fractions containing SSB_BA_ were identified by SDS-PAGE and combined (Fraction IV). The Fraction IV, adjusted to 0.25 g/ml ammonium sulfate, was incubated on ice for two hours that resulted in the selective precipitation of SSB_BA_. The SSB_BA_ precipitate was collected by centrifugation at 43000 x g for 60 min at 0–1°C. The pellet (Fraction V) was resuspended in 10 ml of buffer A_100_. Homogeneity was assessed by SDS-PAGE.

### Assay of SSB biological activity

The standard assay, based on the stimulation of DNA helicase ativity of DnaB protein, was carried out in 1 ml of buffer B containing 1 mM DTT, 25 mM KCl, 3.5 mM ATP and 4.2 nmol of the 55 bp partial duplex substrate containing the following oligonucleotides [51]:

(2) $$\begin{array}{c} 5\text{'}\text{GTCTTTCTGAGTACGAGAGTTCTGAGCAGTTCCAATACATTTTTTTTTTTTTTTT}\left[\text{Cy}5\right]-3\text{'}\\ 5\text{'}\left[\text{Cy}3\right]\text{TTTTTTTTTTTTTTT}\text{ATGTATTGGAACTGCTCAGAACTCTCGTACTCAGAAAGAC}-3\text{'}\text{.} \end{array}$$

Italicized nucleotides denote non-complimentary bases that create the fork structure of the duplex. Fluorescence emission spectra of the samples, before and after reaction, were recorded between 550–750 nm with 519 nm excitation with 8 nm slit-width. Reaction was initiated by adding 0.5 μg/ml DnaB_BA_ helicase to the reaction mixture and incubated for 15 min at 37°C and FRET was measured. SSB_BA_ (3 μg/ml) was added to the reaction mixture where indicated. DnaB_BA_ helicase unwinding of the duplex led to inhibition of the FRET between Cy3 and Cy5. SSB_BA_ was required for efficient helicase action of DnaB_BA_ which was the basis of the assay. By using native and heat denatured substrates, it was determined that 1% decrease in FRET is equivalent to 3 pmol DNA unwinding in terms of base pairs (bp).

### Steady-state fluorescence measurements

Fluorescence anisotropy was measured to investigate DNA binding by SSB_BA_ in solution [40,52]. Fluorescence measurements were carried out using a steady-state-photon counting spectrofluorometer, PC1 with Vinci software, from ISS Instruments (Champaign, IL) and Fluoromax4-TCSPC with time-resolved fluorescence from Horiba Instruments Inc. (Edison, NJ). Excitation and emission slits were adjusted to 8 nm to maximize intensity counts [53]. Temperature during measurements was maintained using a programmable Peltier-controlled cuvette holder from Quantum Northwest Inc. (Seattle, WA).

### Fluorescence anisotropy analysis of equilibrium ssDNA binding

The oligonucleotide was diluted to a concentration of 1 nM and titrated with SSB_BA_ within a concentration range of 0.1 nM to 1μM. The sample was incubated for 2 min after each addition and thermostated at 25°C. Anisotropy measurements were carried out in general as described above except the excitation wavelength was 480 nm and emission anisotropy was measured at 540 nm. The standard deviation for the anisotropy values was <5 mA. Anisotropy at each titration point was measured three times for 10 s. and averaged. The total fluorescence intensity did not change significantly (≤10%) with increase in SSB_BA_ concentration. Therefore, fluorescence lifetime changes, or the scattered excitation light, did not affect the anisotropy measurements.

(3) $$\text{Anisotropy}\text{,}\;\text{A}\text{,}\;\text{is defined as}:\text{A}=\left({\text{I}}_{\text{vv}}–\text{G}\times {\text{I}}_{\text{vh}}\right)/\left({\text{I}}_{\text{vv}}+2\times \text{G}\times {\text{I}}_{\text{vh}}\right)$$

where, G is the instrumental correction factor for the fluorometer and it is defined by

(4) $$\text{G}={\text{I}}_{\text{hv}}/{\text{I}}_{\text{hh}}$$

I_vv_, I_vh_, I_hv_ and I_hh_ represent the fluorescence signal for excitation and emission with the polarizers set at (0^0^, 0^0^), (0^0^, 90^0^), (90^0^, 0^0^) and (90^0^, 90^0^) respectively.

The interaction of SSB_BA_ with the labeled oligonucleotide can be represented as follows:

(5) $${\text{SSB}}_{\text{BA}}\;\left[\text{P}\right]+\text{ssDNA}\;\left[\text{R}\right]⇆{\text{SSB}}_{\text{BA}}\cdot \text{ssDNA }\left[\text{RP}\right]$$

Where, R is the ligand i.e., labeled oligonucleotides and P is SSB_BA_.

At equilibrium, K_A_, the equilibrium association constant can be given as

(6) $${\text{K}}_{\text{A}}=\left[\text{RP}\right]/\left[\text{R}\right]\;\left[\text{P}\right]$$

(7) $${\text{K}}_{\text{A}}\left[\text{R}\right]\;\left[\text{P}\right]=\left[\text{RP}\right]$$

Fraction of the binding sites occupied, *f*, can be represented as

(8) $$f=\left[\text{occupied binding sites}\right]/\left[\text{total binding sites}\right]=\left[\text{RP}\right]/\left(\left[\text{R}\right]+\left[\text{RP}\right]\right)$$

Substituting for [RP] and rearranging the equation we get

(9) $$f={\text{K}}_{\text{A}}\cdot \text{P}\;/\;\left(1+{\text{K}}_{\text{A}}\cdot \text{P}\right)$$

(10) $$f=\left[\text{P}\right]\;/\;\left(\;\left[\text{P}\right]+1/\;{\text{K}}_{\text{A}}\right)$$

Similarly, equilibrium dissociation constant K_D_ (K_D_ = 1/K_A_) can be expressed as

(11) $$f=\left[\text{P}\right]/\left(\left[\text{P}\right]+{\text{K}}_{\text{D}}\right)$$

(12) $$\text{Atf}=0.5\text{,}\;{\text{K}}_{\text{D}}=\left[\text{P}\right]$$

Thus, K_D_ can be further defined as the protein concentration at which half of the sites are occupied when ligand concentration is constant, as in the present case. Non-linear regression analysis of the anisotropy plot (anisotropy vs. log[SSB_BA_]) was carried out using Prism 6.1 software (GraphPad Software Inc., San Diego, CA) and the concentration of SSB_BA_ required to bind 50% of oligonucleotides was computed using the following equation:-

(13) $$\text{Y}={\text{Y}}_{\text{MIN}}+\left[\left({\text{Y}}_{\text{MAX}}-{\text{Y}}_{\text{MIN}}\right)/\left(1+10^{\left({\left(\text{LogEC}50-\text{X}\right)}^{{\text{n}}_{\text{app}}}\right)}\right)\right]$$

where, Y_MIN_ and Y_MAX_ are the anisotropy values at the bottom and top plateaus respectively. X represents log[SSB_BA_] (where [SSB_BA_] represents total concentration of SSB_BA_) and X_0_ is the X value when the anisotropy is halfway between the top and the bottom of the plot and n_app_ is the Hill coefficient.

### FRET analysis of ssDNA binding by SSB_EC_ and SSB_BA_

FRET analysis was used to monitor ssDNA binding by SSB as described (22). Reaction mixtures were assembled on ice and incubated at 25°C for 5 min before FRET analysis. Reaction mixtures contained 40 nM labeled Cy5-(dT)_70_-Cy3 oligonucleotide and the indicated amount of SSB_EC_ or SSB_BA_ in a total volume of 1 ml. SSB_BA_ or SSB_EC_ titrations were performed with PC1 spectrofluorometer with the monochromator set at 515 nm for excitation for the Cy3 donor and with the monochromator set at 665 nm emissions for the Cy5 acceptor. Slit width was 8 nm.

## Abbreviations

B. anthracis: Bacillus anthracis; ssDNA: Single stranded DNA; DTT: Dithiothreitol; SDS: Sodium dodecyl sulfate; PAGE: Polyacrylamide gel electrophoresis; rNTP: Ribonucleotide triphosphate; BSA: Bovine serum albumin; SSB: Single stranded DNA binding protein; SSBBA: SSB of Bacillus anthracis; DnaBBA: DnaB helicase of Bacillus anthracis; FRET: Fluorescence resonance energy transfer; mA: Millianisotropy; kDa: Kilo Dalton.

## Competing interests

The author(s) declare that they have no competing interests.

## Authors’ contributions

EB-F was involved in the design of the study, experiments, and manuscript preparation. JK was involved in the design of the study and carried out purification and fluorescence experiments. SB was involved in the design of the study and overall direction of the project. All authors read and approved the final manuscript.
